# Psychosocial transformation and peacebuilding: analysis of the community impact of development and peace programs in Colombia

**DOI:** 10.3389/fpsyg.2026.1733051

**Published:** 2026-10-16

**Authors:** John Edisson Garcia Peñaloza, Nelly Campos Ramirez, Nazli Melissa Mosquera Conde

**Affiliations:** 1 School of Administrative, Accounting, Economic, and Business Sciences (ECACEN) - UNAD, Universidad Nacional Abierta y a Distancia UNAD (Neiva-Huila), Bogotá, Colombia; 2 Social Inclusion for Regional Development and Community Outreach – UNAD, Universidad Nacional Abierta y a Distancia UNAD (Neiva-Huila), Bogotá, Colombia

**Keywords:** armed conflict, community development, community psychology, peacebuilding, resilience

## Abstract

**Introduction:**

The prolonged armed conflict and the impact of its consequences following the signing of the Peace Treaty generated profound psychosocial impacts on Colombian communities. These impacts include the fragmentation of the social fabric, distrust, and limited citizen participation. In this context, the Development and Peace Programs were designed as initiatives aimed at promoting territorial reconciliation and strengthening social capital.

**Objective:**

The objective of this study was to analyze, through the voices of community leaders, the impact of these programs on psychosocial transformation and peacebuilding.

**Methods:**

This study used a multimodal qualitative design grounded in a hermeneutic approach and supported by analytical procedures drawn from grounded theory. Semi-structured interviews were conducted with 17 community leaders with extensive experience in the selected programs. The data were analyzed through open, axial, and selective coding with the support of qualitative analysis software.

**Results:**

The results demonstrate a transition from conditions of vulnerability, isolation, exclusion, and fear to scenarios of social cohesion, citizen empowerment, and strengthening of community mental health. Furthermore, the authors observed that the programs facilitated processes of collective agency and increased critical political participation. Another crucial finding was the impact on the redefinition of traumatic experiences through dialogue, free expression, and trust-building.

**Conclusion:**

The programs supported processes of identity reconfiguration, psychosocial resilience, and the recovery of collective voice in the participating communities.

## 1 Introduction

Colombia persistently ranks among the countries with the highest levels of violence globally, a fact documented by various national and international studies (Gillooly, 2022). This condition, according to the findings of Niño and Palma (2023), is manifested in statistical indicators that reflect a homicide rate and general indicators of violence (political, gender, racial) that are significantly high in relation to its total population. Consequently, the data place the Country in an unfavorable position within the regional context, but also reflect the multidimensionality of violence and its impact on multiple spheres of national life (Pérez-Rincón et al., 2022).

Colombia has experienced one of the longest internal armed conflicts in the Western Hemisphere, spanning more than six decades of confrontation among the State, left-wing guerrilla groups (primarily the FARC-EP and the ELN), right-wing paramilitary groups, and, more recently, criminal bands and dissident factions (Palma, 2023). This conflict has left, as of the count dated August 31, 2026, a total of 10,357,896 victims of the armed conflict, of whom 9,231,020 have been internally displaced persons, making Colombia one of the countries with the highest number of displaced populations worldwide (Unidad para las Víctimas, 2026). The impact has been particularly severe in rural areas, where the historical absence of the State, land concentration, and the presence of illicit economies created conditions conducive to the persistence of violence (Castillo Rojas and Lévy, 2025). Unlike conventional interstate wars, the Colombian conflict has been characterized by its fragmentation, its territorial heterogeneity, and its gradual transformation over time, which has produced differentiated impacts across regions and populations.

The persistence of violence in Colombia is explained by a convergence of structural factors that transcend the armed confrontation itself. These include profound inequality in land tenure, inherited from an exclusionary agrarian model; the fragmented and selective presence of the State, which has generated power vacuums exploited by armed actors; and the consolidation of illicit economies (drug trafficking, illegal mining, and extortion) that finance and perpetuate cycles of violence (Aponte González et al., 2024; Pérez-Rincón et al., 2022). Following the signing of the 2016 Peace Agreement, these dynamics did not disappear but rather reconfigured, manifesting in the systematic assassination of social leaders and territorial defenders, especially in peripheral regions (Samper and Krause, 2024). This structural complexity explains why the transition from armed confrontation to sustainable peace has been neither linear nor uniform, and why territorial approaches to peacebuilding became a central pillar of post-agreement interventions in Colombia.

In this regard, it is crucial to note that in the Colombian scenario, multiple factors converge that explain the persistence of violence, ranging from unequal power relations to the complex geographies of the nation (Aponte González et al., 2024; Edeby and San Sebastián, 2021). However, the internal armed conflict has been a structural element that has permeated all social strata and conditioned the very configuration of national identity and its external projection (Albarracín et al., 2023; Pérez Gamboa et al., 2023). This structural character of the conflict explains why its effects cannot be understood solely through statistical indicators, but require an analysis of the psychosocial and community dimensions that shape the lived experiences of affected populations.

In the Colombian context, the concept of territorial peace represented a paradigm shift compared to traditional, centralized models of peacebuilding. Although conceptually it coexists with proposals such as peace regions or total peace, this approach stems from the recognition that the historical conflict did not affect the entire nation uniformly (Cairo et al., 2024). From this perspective, authors such as Cairo et al. (2024) and Ríos (2023) acknowledge the geographical variation in the impact and intensity of the violence, factors determined by historical dynamics. Kurtenbach (2025), for her part, argues that the actual manifestations of peace, as a normative and territorial process, were conditioned by the analysis of differences, citizen security, and participation. Therefore, territorial peace is not understood as simply the absence of armed confrontation decided in Bogotá. Instead, this concept refers to the bottom-up evolution of a historically insufficient institutional presence, which is not limited to state, military, and paramilitary actors, but also encompasses civil society and its agency.

Communities have been immersed, directly or indirectly, in these dynamics of confrontation as a result of the struggle between various armed actors with divergent ideological orientations, with the civilian population being the most affected by these disputes. As a result, citizens have suffered repression, forced displacement, homicides, kidnappings, and systematic violations of their human rights. Specifically, the study by Samper and Krause (2024) confirms that, despite the signing of the peace treaty in 2016, tensions between the actors involved vary and are marked by tensions caused by land ownership, illegal trafficking, and civil rights.

However, it is imperative to recognize that Colombian communities, while confronting the conflict territorially, also actively pursued their own well-being, transcending the passive reception of external aid and interventions. According to testimonies gathered by Lederach (2023), prior to the implementation of formal peace programs, Colombian populations had developed organic forms of resistance, mutual support networks, and survival strategies that formed autonomous social structures connected by community agency. As Courtheyn (2022) rightly points out, despite the signing of the peace agreement, various peasant communities did not wait for the effects of the agreement imposed from above. However, they actively confronted forced displacement and the violence (armed, political, and structural) perpetrated by guerrillas, the army, and paramilitary groups. This historical agency suggests that the success of subsequent interventions cannot be understood as an achievement *ex nihilo*, but rather as a gradual process of strengthening pre-existing community resources and resilience.

This situation has generated a profound psychosocial impact, given that territorial uprooting produces concatenated problems in the social, economic, and familial spheres. Furthermore, the impact of long-term armed conflicts tends to fracture community ties and expose people to multiple risk factors (Borges Machín and González Bravo, 2022; Jain et al., 2022; Trujillo et al., 2021). Along these lines, Trujillo et al. (2021) assert that the damage transcends the gnoseological diagnoses commonly associated with conflict scenarios, such as anxiety, depression, or post-traumatic stress, since they affect empathy and can manifest in the high rates of multidimensional violence already discussed.

The psychosocial impact of the Colombian conflict has been extensively documented. Multiple studies have shown that communities affected by armed violence face severe limitations in deploying their resources and capabilities, both in rural and urban areas (Ocampo Talero et al., 2022). These limitations are not only a direct consequence of the conflict itself but are also exacerbated by the difficulty of health professionals to offer coherent responses to demands for psychological help (Duarte Cuervo, 2023). Women, in particular, have been exposed to a double victimization: first by the violence, and then by the absence or inefficiency of support structures and the inability of governments to translate discourses and policies into concrete practices (Gómez-Cano et al., 2024).

Specifically, the psychological effects of armed violence manifest in problems in social ties, characterized by permanent feelings of fear and anxiety that impede participation in collective construction processes. Fear and anxiety, although initially adaptive responses to threatening situations, when chronically prolonged in contexts of armed conflict, generate dysfunctional identities where emotions are repressed, hindering the construction of relationships that promote individual and collective well-being (Moreno-Chaparro et al., 2022). The specialized literature corroborates that these traumatic experiences of an emotional and relational nature often face serious difficulties in their functional processing, and that difficulties in emotional regulation may be due to the systematic use of avoidance as a predominant coping strategy (Jain et al., 2022). This inadequate management of emotions related to trauma and daily demands is deepened by the absence of specialized psychosocial care in populations of victims of the conflict (Duarte Cuervo, 2023; Gómez-Cano et al., 2024; Pérez Gamboa et al., 2023).

From the perspective of community psychology, the ability to exercise the right to free expression within a context of respect for fundamental rights constitutes a critical factor in the recovery of the social fabric (Sanabria Martínez, 2022). Therefore, community participation and empowerment processes assert the role of individuals and groups in proposing concrete steps to strengthen their well-being (Moreira and Reis Fonseca, 2024). In this sense, psychological well-being is a complex category that cannot be reduced to the presence or absence of symptoms, but is instead a construct related to other fundamental concepts such as stress, coping, resilience, and social support (Pérez Valdivia et al., 2024). By recovering their leading role in the face of external factors, communities develop their capacity to understand situations and find the collective meaning that drives change in the face of phenomena such as fear, apathy, and loss of cohesion. This is why the programs acted as facilitators of reconciliation processes by promoting functional coexistence, truth, and forgiveness (Pérez Gamboa et al., 2023).

The severity of these circumstances is crucial for shaping a coherent national project. In line with previous paragraphs, international evidence warns that, in contexts of armed conflict, a significant percentage of people exposed to traumatic events develop severe mental disorders (Bendavid et al., 2021; Moreno-Chaparro et al., 2022). In contrast, another segment, not always addressed or rendered invisible in typical diagnoses, exhibits behaviors that limit their effective functioning in the community. These conditions substantially alter the ability to unite the members of a nation and, in a systemic effect, reproduce scenarios of violence that perpetuate themselves over time.

Given these realities, the Development and Peace Programs (PDPs) emerged in Colombia as a distinctive model of civil society-led peacebuilding. Farnsworth-Alvear (2019) and Galindo Villarreal (2020) trace the trajectory of these programs from the rural rehabilitation initiatives of the National Front (1958–1962) to the territorial programs following the 2016 Agreement, evidencing a research continuity of more than six decades. Within this framework, the Magdalena Medio Development and Peace Program (PDPMM), founded in 1995, stands as the most emblematic and studied experience of this tradition. Katz (2004), who served as deputy director of the program, describes the PDPMM as an effort to build peace based on two interrelated endeavors: a dynamic of regional socioeconomic development that positively impacts the poorest inhabitants, and a process of reconstruction and recovery of collective goods, beginning with access to the State, education, health, the environment, and cultural heritage.

The present study focuses on three PDPs that operate in distinct socio-territorial contexts: the Magdalena Medio Development and Peace Program (PDPMM), founded in 1995 (Katz, 2004; Barreto Henriques, 2015); the PRODEPAZ Development and Peace Program in eastern Antioquia, founded in 1999 (Molina Sáenz, 2008; European Union Delegation to Colombia, 2011); and the Huila and Amazonian Foothills Development and Peace Program (HUIPAZ), founded in 2004 (Cano et al., 2020). While these programs share a common institutional framework and a commitment to territorial peacebuilding, they differ in their historical trajectories, territorial reach, and the specific dynamics of the armed conflict in each region. These differences are described in detail in the Research Context section (Section 2.1), which provides the socio-territorial characterization of each program and the criteria for their selection as case studies.

Barreto Henriques (2015) evaluates the PDPMM as a consolidated “peace laboratory” and a valid model of positive peacebuilding at the regional level, from which lessons and good practices can be drawn for a post-conflict scenario. This tradition has been continued by research such as that of Jaramillo-Grajales and Cárdenas-Vásquez (2021), who analyze the characteristics of psychosocial intervention in the PDPMM as a peacebuilding scenario, and that of Díaz et al. (2021), who examine the critical lessons of Colombian territorial peace. Likewise, the evaluation of the success of these programs has been approached from various perspectives: Carranza-Franco (2019) analyzes the dilemmas of DDR (Disarmament, Demobilization, and Reintegration) processes, Nilsson and Marín (2021) evaluate the obstacles of the National Comprehensive Program for the Substitution of Illicit Crops (PNIS), Galindo Villarreal (2020) examines the reconciliatory potential of the Development Programs with a Territorial-Based Approach (PDET), and Miklian (2020) studies the role of the business sector in local peacebuilding. Finally, the work of Borja et al. (2026) constitutes a fundamental contribution to this tradition, as it analyzes how Colombian sociology has interpreted violence and peace since the mid-twentieth century, highlighting the role of the Sociology Circle of the National University and the peace commissions in the construction of knowledge about the conflict.

As this review of the literature demonstrates, this body of literature has consistently highlighted the PDPs’ capacity to foster participatory processes and to articulate local development initiatives with peacebuilding objectives. However, much of this research has focused on institutional design, territorial coverage, or economic outcomes, and has relied primarily on documentary analysis or program evaluations. What remains underexplored is the psychosocial dimension of these programs, in relation to how they have transformed the subjective and relational conditions of communities affected by violence, from the perspective of those who have led these processes on the ground. Given this, we adopted as our guiding research question: What has been the impact of the Development and Peace Programs from the perspective of the communities involved?

This gap is significant because the broader field of peacebuilding has increasingly recognized that sustainable peace cannot be achieved solely through institutional agreements or economic development, but requires the transformation of the psychosocial conditions that sustain violence and exclusion. In community psychology, scholars have long argued that collective well-being, social cohesion, and the recovery of voice are central to the reconstruction of social fabric in contexts affected by political violence (Martín-Baró, 1994; Lykes, 2001; Sanabria Martínez, 2022; Moreira and Reis Fonseca, 2024). Similarly, peace psychology and transitional justice studies have emphasized the importance of processing traumatic experiences, rebuilding trust, and fostering collective agency as prerequisites for reconciliation (Lira, 2010; Pérez Valdivia et al., 2024; Pérez Gamboa et al., 2023). International peacebuilding scholarship has also increasingly emphasized the need to move beyond top-down, externally driven interventions toward approaches that recognize communities as active agents of their own transformation (Lederach, 1997; Mac Ginty, 2014; Autesserre, 2021; Kurtenbach, 2025). Yet, empirical research that documents how these processes unfold in practice, particularly in contexts where communities have been both victims of violence and active agents of their own transformation, remains limited.

This study addresses that gap by analyzing the psychosocial impact of the PDPs from the perspective of community leaders in three regions of Colombia. Rather than assuming a linear transition from vulnerability to empowerment, it explores the tensions, contradictions, and persisting challenges that defined the reality of peacebuilding in the Colombian context. In doing so, it contributes to ongoing debates in community psychology, peace psychology, transitional justice, and Latin American studies in three ways. First, it offers empirical evidence on how community-based peacebuilding programs can foster psychosocial transformation in contexts of prolonged violence. Second, it provides a situated analysis of the mechanisms (trust-building, free expression, collective agency, and political participation) through which these transformations occur. Third, it highlights the limitations and challenges of externally driven interventions, offering lessons that may be relevant for peacebuilding initiatives in other post-conflict settings.

## 2 Methodology

The research was structured, in accordance with Pérez Gamboa et al. (2024), under a multimodal qualitative methodological approach, combining hermeneutic assumptions and analytical procedures from grounded theory. In addition, it had a design oriented to understanding the specific impact on strengthening community mental health of the initiatives implemented as part of development and peace programs. To this end, the study was epistemologically grounded in the hermeneutic approach, since we recognized that the specificity of human phenomena is not always reducible to quantifiable or objective explanations in the positivist sense.

Specifically, reality is conceived as a culturally situated and constructed process, where, rather than obeying universal laws, it is subject to the lived experiences and emotional universes of the participants. Supported by the arguments put forward by Pérez Gamboa et al. (2024) on the nature and purposes of qualitative research methodology, the choice of this route was due to its capacity to favor the in-depth exploration of both development and peace programs and the configurations of collective mental health. The research was thus oriented toward the interpretation of the diverse ways of inhabiting the lifeworld, prioritizing the understanding of human actions from the meanings constructed by the social actors involved in the processes of the Development and Peace Programs.

### 2.1 Research context

Peace and Development Programs (PDPs) are civil society organizations that facilitate processes aimed at strengthening social capital, promoting the construction of territorial peace, and improving dignified living conditions in the communities they influence (Rettberg and Dupont, 2025). This conceptualization allows us to understand the historical relevance these initiatives have had for Colombian communities, particularly during periods marked by the prevalence of armed conflict as a common denominator across vast national territories.

The three PDPs analyzed in this study operate in distinct socio-territorial contexts and were selected based on five substantive criteria: (a) regional representativeness, as they operate in regions with differentiated conflict dynamics, institutional presence, and socio-economic conditions; (b) organizational trajectory, as all three have been operating for more than a decade and are part of the National Network of Development and Peace Programs (Redprodepaz); (c) articulation with the Peace Laboratories, as all three have been strategic partners of the Peace Laboratories financed by the European Union; (d) institutional accessibility, as all three facilitated access to community leaders and provided databases of participants; and (e) socio-territorial diversity, as they operate in regions with different levels of conflict intensity, ethnic composition, and economic vocations. Although logistical proximity to the researchers facilitated access to the three programs, this criterion was not the primary basis for their selection.

#### 2.1.1 PRODEPAZ development and peace program

In the eastern region of Antioquia, PRODEPAZ was created in September 1999 through a joint initiative of social organizations with diocesan institutional support (Diocese of Sonsón-Rionegro and Diocese of Barrancabermeja) and private sector entities (ISA, ISAGEN, PROANTIOQUIA, CINEP). Its mission focuses on working with vulnerable communities affected by various social phenomena, with special emphasis on populations victimized by the armed conflict in rural and urban settings (European Union Delegation to Colombia, 2011; Cano et al., 2020).

PRODEPAZ’s initial actions focused on promoting projects aimed at strengthening human, social, and economic development, creating spaces for citizen engagement and participation at a time when war-torn dynamics severely hampered communities’ ability to build collective alternatives. The program has distinguished itself by creating community-based settings that facilitate the construction of development alternatives, highlighting productive initiatives that allow the population to market and access locally grown or manufactured goods, along with training processes aimed at strengthening civic capacities. Its operational strategy is articulated through four lines of action: construction of conditions for peaceful coexistence and dignified life; promotion of organizational and institutional strengthening; articulation with territorial planning processes; and development of productive and commercial initiatives. Its territorial coverage includes 23 municipalities in the subregions of Altiplano, Embalses, Bosques, Páramos, and Porce-Nus, covering an area of approximately 8,785 km^2^ (European Union Delegation to Colombia, 2011; Cano et al., 2020).

#### 2.1.2 Huila and Amazonian Foothills Development and Peace Program (HUIPAZ)

In response to the dynamics and impacts generated by the armed conflict in the department of Huila, HUIPAZ emerged in 2004 as part of the National Network of Development and Peace Programs (Redprodepaz). The fundamental mission of this program has been to contribute to the consolidation of alternatives to poverty and the effects of regional violence. To this end, its approach has emphasized promoting a sociocultural transformation that fosters territorial and community mobilization for peace, establishing as its pillars the deepening of democratic values and the construction of a contextualized model of sustainable human development (Cano et al., 2020).

HUIPAZ operates through strategic alliances and co-management mechanisms at multiple levels of governance and in neighboring municipalities. Its operational strategy is articulated through seven lines of action: land and rural development; peacebuilding; peace observatories; territorial nodes; knowledge management; human rights; and a dialogue platform known as the “Network of Residents” (Red de Pobladores). Its territorial coverage includes 37 municipalities in the department of Huila and some municipalities in Caquetá (Cartagena del Chairá, Paujil, Puerto Rico, and Morelia) and Tolima (Ortega, Alpujarra, and Chaparral) (Cano et al., 2020).

#### 2.1.3 Magdalena Centro Peace Development Program (PDPMC)

In a context marked by the persistent presence of various illegal armed groups in the region, PDPMC emerged in 1995 in coordination with multiple sectors, institutions, and businesses in the Magdalena Centro region. This civil society initiative was established as a platform to create enabling conditions for endogenous development and the formation of spaces for peaceful coexistence for residents and communities affected by the historical conflict (Katz, 2004; Barreto Henriques, 2015).

The distinctive feature of this initiative is its dual nature, as it acts both as a corporate entity and as a psychosocial and community intervention program. In the first case, PDPMC seeks to facilitate community empowerment processes that serve as a basis for residents to take ownership of their development and contribute to peacebuilding. In the second case, the program has promoted the formulation and implementation of autonomous and inclusive proposals that allow communities to exercise their rights and exploit their inherent capabilities. Its operational strategy is articulated through three strategic lines: culture of peace and reconciliation; integral sustainable development; and infrastructure, environment, and society. These are complemented by two transversal lines: governance and governability; and communication and regional learning, supported by a dialogue platform known as the “Subregional Working Tables” (Mesas Subregionales de Trabajo) (Katz, 2004; Barreto Henriques, 2015).

PDPMC operates in the Magdalena Centro region, which comprises 17 municipalities across four departments (Caldas, Cundinamarca, Boyacá, and Antioquia), with its headquarters in La Dorada (Caldas). The region is characterized by its strategic location along the Magdalena River, its agricultural vocation (coffee, sugarcane, livestock), and its history of armed conflict and illicit economies (Katz, 2004; Barreto Henriques, 2015).

### 2.2 Sampling and participants

The study focuses on three PDPs that operate in distinct socio-territorial contexts: PRODEPAZ in eastern Antioquia, HUIPAZ in Huila and the Amazonian Foothills, and PDPMC in the Magdalena Centro region. The selection of these three cases followed a purposive sampling strategy based on five criteria (Bouncken et al., 2026): regional representativeness; organizational trajectory; articulation with the Peace Laboratories; institutional accessibility; and socio-territorial diversity. Although logistical proximity to the researchers facilitated access to the three programs, this criterion was not the primary basis for their selection.

#### 2.2.1 Participant recruitment and sampling

The recruitment of participants followed a two-stage process, consistent with the principles of purposive sampling in qualitative research (Bouncken et al., 2026). In the first stage, the research team identified the three programs based on the criteria described above. In the second stage, we employed a purposive sampling strategy to select community leaders who met three inclusion criteria, following the recommendations for criterion-based sampling in qualitative research (Nyimbili and Nyimbili, 2024): at least 5 years of continuous involvement in processes promoted by the PDPs; documented knowledge of the programs’ lines of action and operational mechanisms; and recognized leadership roles within their communities, verified through community recognition and participation in local governance spaces (e.g., Community Action Boards, victims’ roundtables, youth platforms, or peace councils).

To avoid the risk of institutional bias in the selection of participants, we requested from each program a broad list of leaders who met the inclusion criteria. We verified the leaders’ trajectories through independent sources, including community records, local government databases, and public documents, to ensure that their leadership roles were not solely dependent on their relationship with the program. Finally, we explicitly sought to include leaders with diverse perspectives, including those who had expressed criticisms or reservations about the programs, as well as leaders from communities that had not benefited equally from the interventions. This strategy allowed us to capture a range of experiences that goes beyond the institutional narrative of success.

Participation was voluntary and was confirmed through signed informed consent. Before each interview, participants received information about the study’s objectives, scope, and planned use of the findings. The final sample included 17 participants between 18 and 64 years of age from municipalities located within the programs’ areas of influence. Most had completed secondary education and were actively engaged in community leadership alongside other occupations.

In order to safeguard the confidentiality of the individuals participating in the semi-structured interviews, a coding system was used for their identification throughout the description, argumentation, and analysis. Table 1 shows the system of abbreviations used for coding the verbalizations, which will facilitate the understanding of the data presented in the results section.

**Table 1:** System of acronyms for coding verbalizations.

F/M	Gender of the participant. (F) Female (M) Male
#	Age of the participating person
PDPMC	Magdalena Centro Peace Development Program (PDPMC)
PDPHP	Huila and Amazonian Foothills Development and Peace Program (HUIPAZ)
PDP	PRODEPAZ Development and Peace Program
Example of encoding	F58PDPMC F49PDPHP M25PDP

### 2.3 Information collection

The research process began with an exhaustive documentary review that allowed us to delimit the relevant thematic axes to approach the phenomenon under study. Although according to Pérez Gamboa et al. (2024), the role of documentary review in qualitative research has been disputed or even denied due to its inductive nature, we accepted their assessment, indicating that this type of methodological exercise makes it possible to identify previous research and conceptual foundations for the construction of reference frameworks and data collection instruments.

This analysis identified research precedents that used semi-structured interviews as the primary method of data collection in communities affected by the armed conflict. Consequently, we designed an interview guide to collect data at both the descriptive and in-depth levels, a consideration based on the conversational nature of this type of interview. To this end, we used the narrative method, which posits the importance of interpreting participants’ discourse. The categories and lines of interest identified *a priori* facilitated a situated analysis, but also the emergence of complex experiences related to the phenomenon under study.

Due to the contingencies generated by the pandemic, the interviews were conducted by telephone, adhering to both biosafety recommendations and the ethical parameters established for research in health emergency contexts, including obtaining informed consent from participants. The fieldwork extended from March 2020, with the beginning of informed consent requests and initial contacts, until February 2022, when the authors conducted final post-interview checks. We implemented a semi-structured protocol for the interviews, consisting of self-developed, predominantly open-ended questions that addressed the fundamental research themes represented in each of the analysis categories. The interviews lasted approximately 60 min, but participants also used short audio recordings and text messages to answer specific questions, reinforce particular narratives, or provide concrete examples.

The interviews were conducted and transcribed in Spanish, ensuring that their processing accurately reflected the linguistic, social, and cultural nuances. The authors translated the participants’ verbalizations, and the grammar was reviewed using Grammarly. Additionally, authors commissioned a professional translator to review the final version of the text to verify the accuracy of the translation.

### 2.4 Systematization and analysis strategy

We based the methodological approach for data analysis on the analytical procedures of grounded theory (Charmaz and Thornberg, 2020; Pérez Gamboa et al., 2024). This type of design represents an alternative approach to human realities, as it allows for the generation of interpretive frameworks based on observed phenomena. Because the purpose of the research was not to generate a substantive theory, as is often the case in “pure” grounded theory designs, the authors selectively adopted conceptual and procedural elements of this methodological framework, which were adjusted to the problematic situation and the objectives of the study.

We developed the analytical process through sequential stages that included full-text transcription of the information, microanalysis of the content, open coding, axial coding, and selective or comprehensive coding. The initial stages corresponded to the descriptive-analytical process, while the subsequent stages constituted the interpretive-synthetic process (Charmaz and Thornberg, 2020; Pérez Gamboa et al., 2023). We carried out the analysis by processing the data using specialized qualitative research software.

In the initial phase, we compiled audio recordings from interviews with leaders from the study territories. Subsequently, we fully transcribed the narrative information, a process that included assigning confidential identification codes to each of the participating actors, thus ensuring the ethical principles of confidentiality and anonymity. This phase facilitated the identification of significant expressions and the discursive intentions of the social actors.

The microanalysis consisted of a thorough, line-by-line examination of each of the transcribed texts that comprised the hermeneutical units (Charmaz and Thornberg, 2020; Pérez Gamboa et al., 2023). This exercise allowed for a specific examination of the data obtained and an understanding of their substantive relevance to the research. As a result of this stage, fundamental hermeneutical units were organized, based on the initial categories that allowed for establishing relationships between the different concepts emerging from the discourses of the social actors interviewed.

Open coding aimed to identify the fundamental concepts related to the research and to uncover the substantive dimensions of the phenomenon under study (Charmaz and Thornberg, 2020; Pérez Gamboa et al., 2023). During this stage, the researchers maintained a constant openness to emerging ideas from the textual data, which allowed the coding to reveal the natural direction of the study based on the participants’ voices.

We understood axial coding as the process of regrouping data that were fragmented during open coding (Charmaz and Thornberg, 2020; Pérez Gamboa et al., 2023), allowing for the establishment of meaningful relationships between the categories identified in the previous phase. This stage allowed for the identification of core categories based on the narratives expressed by the leaders, facilitating the analysis of the impact of development and peace programs through a systematic process in which semantic networks emerged that were consistent with the categories guiding the research.

Selective coding represented the final phase of the analysis, where we integrated concepts into core categories, and completed those requiring further development and refinement. This stage allowed the researchers to unify the central idea emerging from the significant elements related to the research object into integrative categories. The final comprehensive text was constructed based on the systematization carried out in the previous phases, with an interpretive reading that integrated the relevant elements, leading to a comprehensive understanding of the phenomenon under study and allowing for an answer to the research question posed.

### 2.5 Supporting the validity of the study

Regarding scientific validity in qualitative research, researchers achieve it by contrasting and interpreting findings until they reach understanding and consensus. Following this general principle, we subjected the findings from fieldwork and coding to a rigorous process of methodological triangulation with the results of previous research.

The importance of combining the inductive generation of categories with the constant and simultaneous comparison of all observed social incidents supported the validation and reliability of our methodological approach. This systematic approach to data analysis ensured that we continuously reviewed emerging elements throughout the process. Additionally, it is significant to highlight that the researchers carried out a sequence of critical reflection, which allowed them to creatively relate themes and respond to the observed reality in a process that was constantly changing.

Due to the complex nature of research in a post-conflict context, the authors implemented a trauma-informed approach to prevent re-victimization and ensure a dialogue focused on empowerment and the analysis of the transformations fostered by the programs. Furthermore, potential biases were considered in both the conduct of the interviews and the data analysis. Therefore, attention was paid to managing the researchers’ self-referential knowledge, the potential influence of the researchers on participants’ responses, and the importance of ensuring that the findings remained grounded in the experiences narrated by the participants.

## 3 Results

The systematic organization of the narratives through descriptive categories allowed for the identification of significant trends regarding the impact of the Development and Peace Programs. The analysis revealed substantial transformations in the psychosocial makeup of the communities studied, manifested in a clearly definable contrast between the conditions prior to the program’s intervention and the subsequent reality.

In order to faithfully represent this transition and how it was represented by the participants, the initial analysis reflects on both instances and describes the main elements that characterized them. We presented the integrative analysis, articulated through the main emerging themes and theoretical concepts emerging from the documentary study.

### 3.1 Previous context: vulnerability, isolation, and widespread fear

Before participating in the Development and Peace Programs, people experienced living conditions that prevented them from achieving emotional balance and well-being, significantly limiting the development of their personal and community resources. In relation to these individual and community realities, the leaders interviewed described an evident absence of institutional statehood. This generated not only a real power vacuum but also shared perceptions of abandonment by government entities, which reinforced the state of precariousness and fostered the development of a favorable environment of lack of protection and structural exclusion.

In this sense, the testimonies revealed a network of experiences of helplessness and collective submission that severely restricted individual and group autonomy due to the dynamics imposed by the actions of armed actors. As an expression of this, participants described how they and their communities experienced permanent states of anxiety, marked by constant fear. As was evident, these conditions fostered the creation of a harmful psychosocial climate characterized by deep levels of relational mistrust that shaped distant social relationships.

It is important to clarify that this section describes preexisting community participation, that is, the forms of collective organization and citizen involvement that existed prior to or alongside the PDPs (mainly through Community Action Boards (JACs), religious groups, and mutual aid networks) and not participation in the programs themselves, which is analyzed in Section 3.2.

These relationships, characterized by fear and mistrust, generated significant stagnation in the economic and social development of the territories, a fact that aggravated the direct impact of the conflict in other dimensions. According to the voices of the participants and the experiences they recounted, this preexisting community participation was determined by fear, coercion, a sense of powerlessness, and the disruption of cultural traditions due to the impact of the conflicting forces. The following statements represent these experiences and illustrate the initial state of community participation in local spaces and its emotional impact. When referring to their involvement in preexisting community spaces, the leaders described how fear shaped it:

- *Participation was low because people were afraid of being associated with ideologies or movements that were contrary to the interests of the armed groups*. M39PDPMC
- *Moreover, they participated at the time because there was an armed group that said, “Well, you have to participate, or else we will come for you*.*”* F40PDPHP
- *Not everyone talks to everyone else because of the fear of being associated with armed groups*. F25PDP
- *Armed groups that generated* “intimidation and anxiety” *in community dynamics, but at the time, it was completely coercive; there was no way to speak or express oneself, just* “shut up and keep going.” F40PDPHP

This environment significantly constrained preexisting community participation, which leaders described as weak and progressively eroded. Involvement in community affairs was often reduced to the pursuit of individual needs rather than collective interests. According to the interviewees, this pattern contributed to decision-making processes with limited transparency and weak social legitimacy.

Furthermore, it is concluded that at this stage, people described themselves as victims marked by deep, unprocessed pain, which was aggravated by the lack of understanding and emotional resolution that community relations and institutional support could have provided. However, particular forms of micro-resistance also emerged, demonstrating community agency and the pursuit of well-being through collective action and the maintenance of a sense of normalcy, primarily through the Community Action Boards (JACs), which are legally recognized, democratically elected grassroots organizations that have been the backbone of Colombian community participation since the 1950s. JACs operate at the neighborhood or village level, manage small-scale community projects, and serve as interlocutors with local governments (Castro, 2021). These organizations constituted one of the few legitimate spaces for collective action that remained available to communities during the armed conflict, and community leadership. This orientation towards resilience at the personal level can be observed in these statements from the leaders, reflecting the presence of complex conditions and the transition towards building territorial peace from the historical agency of the inhabitants of these communities:

- *My entire process of becoming a community leader began in the Community Action Board, from being a secretary to becoming the legal representative of the community.* F43PDPMC
- *My activities were those of a typical housewife who made time to go to school and attend Community Action Board meetings.* F60PDPMC
- *I have always felt like a leader; I have been involved in supporting children’s soup kitchens and the Community Action Board.* F60PDPMC

### 3.2 Post-intervention transformation: accompaniment as a catalyst for psychosocial change

The analysis of the narratives revealed substantial transformations in community mental health following the intervention of the Development and Peace Programs. These transformations did not occur as an automatic consequence of the programs’ presence, but through an identifiable sequence of mechanisms: the creation of safe spaces for emotional expression, the progressive rebuilding of trust, the strengthening of organizational capacities, and the subsequent exercise of collective agency in public affairs. The following subsections present this sequence, anchored in the testimonies of the leaders and in the specific activities through which it unfolded.

Community leaders unanimously offered a positive characterization of the impacts generated by the intervention in their territories. This positive assessment was not expressed in abstract terms but was linked to concrete experiences of accompaniment and training. As one leader from Huila stated: “*Before, we didn’t even know how to write a project. The program taught us to identify our own needs, to prioritize them, and to present them to the municipality. That changed everything, because we stopped waiting for someone to come and solve things for us*” (M52PDPHP). The leaders confirmed that the initiatives succeeded in encouraging the formation of new relational dynamics, expressed in greater social cohesion and the emergence of renewed perspectives on peacebuilding. These new dynamics materialized in specific spaces promoted by the programs, such as the subregional working tables (*Mesas Subregionales de Trabajo*) in Magdalena Centro, the youth platforms in Antioquia, and the Network of Residents (*Red de Pobladores*) in Huila. A leader from Magdalena Centro described this shift: “*In the working tables we learned to sit down with people we used to see as enemies, or at least as strangers. At first it was very difficult, nobody wanted to talk. But little by little we began to recognize each other as neighbors with the same problems. Today we work together on the same projects*” (F58PDPMC). These community transformations occurred in such a way that community interactions shifted toward a collective and group-oriented approach to decision-making. This shift was particularly visible in the way communities began to plan and manage productive and social projects collectively, rather than individually. A leader from Antioquia illustrated this: “*Before, each family thought only about its own plot. Now we meet in the Community Action Board and decide together what we are going to do with the resources, which roads to fix, which families need more support. It is no longer every man for himself*” (M47PDP).

Another crucial achievement identified through the narratives was the development of free expression as a psychosocial achievement, in contrast to the fear and mistrust that, as previously mentioned, characterized interactions. The programs facilitated this process through workshops on emotional expression, memory, and conflict transformation, in which participants could verbalize experiences that had remained silenced for years. As one leader explained: “*For the first time we had a space where we could talk about what happened without fear. We cried, we remembered, we wrote. That was very hard, but also very healing. After that, we were able to look at each other differently*” (F49PDPHP).

Thus, the programs acted as catalysts for trust and fostered empowerment processes that led to a new model of community development, characterized by a reflective approach, the rational and collaborative management of conflict situations, and a new understanding of the value of life and human dignity. This “new model” was not a theoretical construct but a set of concrete practices: community-led diagnostics, participatory budgeting exercises, conflict mediation committees, and the systematization of local knowledge. A leader from Magdalena Centro described how conflicts began to be managed: “*Now, when there is a problem between two families or two organizations, we don’t go to the armed group or to the mayor’s office to solve it. We sit down in the working table, we listen to each side, and we look for a solution together. Sometimes we don’t reach an agreement, but at least we don’t kill each other”* (M39PDPMC).

These elements influenced the development of a will for collective construction, reflected in the qualitative transformation of participation in the PDPs and the subsequent exercise of community agency, which came to be characterized as active, committed, and effective. In the words of M39PDPMC: “*Now we are an entrepreneurial, proactive community that is participatory and, above all, I would say, resilient. No obstacle is too big for us; we almost say that every time we encounter a difficult challenge, it motivates us even more and strengthens us, and we say: we’re going to achieve it, and we want to succeed. Thanks to these experiences, we now want to share these tools and strategies to achieve what we set out to do…*”

On the other hand, the interviewees stated that the programs significantly influenced the communities’ citizen and political participation through training processes that equipped them with tools for autonomous decision-making, free from political co-optation and with full awareness of their collective and future implications. These training processes included leadership schools, human rights workshops, and advocacy training that enabled communities to engage with municipal and departmental authorities. A leader from Antioquia described the change: “*Before, we didn’t know how to talk to the mayor. We were afraid, or we thought it was useless. The program taught us how to prepare a proposal, how to ask for a meeting, how to follow up on commitments. Now we sit at the table with the authorities and negotiate, not beg*” (M25PDP). This new approach to political influence was made possible through training and collective knowledge-building opportunities that allowed them to regain the capacity to recognize and claim citizen rights, especially those violated by the armed conflict. In this regard, M25PDP points to the transformations encouraged within the participatory framework promoted by the programs: “*One achievement of these programs was promoting participatory processes aligned with the real needs of the communities through the youth platform. For example, we have led collaborative processes with different population sectors regarding decision-making and advocacy on territorial issues related to human rights, equality, coexistence, and participation*”.

Based on these substantial changes, program participants gradually achieved a new meaning for their traumatic experiences and began to base their identity reconfiguration on their roles as key players in shaping their own realities. This identity transformation was described by the leaders as a shift from seeing themselves as passive victims to recognizing themselves as subjects with rights and capacities. As one leader expressed: “*For many years I said I was a victim, and I felt only pain. Today I say I am a leader, a woman who has walked through fire and came out with something to give to others. That is not forgetting; it is transforming*” (F60PDPMC). When exploring this identity transformation and its implications for behavior, the leaders reported that restoring the voices of those who had been victims of the conflict demonstrated to the communities that verbalizing their experiences facilitated awareness of their own capabilities. Throughout the intervention, these processes strengthened individual and collective resources through mechanisms of mutual support and collective influence. A leader from Huila summarized this: “*The program did not give us the answers. It gave us the space and the tools to find them ourselves. That is the difference. Now we know that we can*” (F40PDPHP).

Finally, it is important to emphasize that, for the leaders, as an expression of the feelings of their communities, peace represented a process of successive construction that integrated material and symbolic elements, but was also inextricably linked to the social and productive development of the territories. This conception of peace was expressed in concrete initiatives such as community productive projects, the recovery of collective memory, and the construction of spaces for dialogue with former combatants in some territories. As one leader stated: “*Peace is not only that they stop shooting. Peace is that we have a road, a school, a health post, and that we can sit down and talk without fear. Peace is built every day, with work*” (M52PDPHP). As can be seen, this conception of peace transcends its vision as a state of absence of violence. It incorporates the possibility for actors to be recognized and transform their environment.

The stakeholders recognized that among the programs’ most significant contributions were technical support, organizational strengthening, support for subregional workspaces, and training in conflict management and resolution skills. Other key contributions included raising awareness about sociopolitical realities and the creation of community spaces that facilitated communities’ involvement in addressing everyday needs, a fact reflected in the consolidation of sustainable territorial transformation processes. However, the leaders also emphasized that these contributions were not uniform across territories, and that their sustainability depended on the continuity of institutional support and on the communities’ own capacity to maintain the processes over time.

Despite acknowledging the numerous contributions made by the programs and their multidimensional impact, the interviews revealed narratives where some leaders recognized that, despite the achievements, specific sectors of the communities had developed a dependence on the programs for managing initiatives or resolving problems. As an example of this dependence in addressing issues where the community does not perceive itself as “*strong*” or “*capable*,” participants stated, “*Sometimes I think some are very dependent… they turn to the program for support on issues where they don’t feel so strong*” (F43PDPMC). However, F43PDPMC also acknowledged that “*some haven’t yet managed to implement changes, while others have already successfully engaged in this management process with regional governments and ministries*,” a testimony that offers valuable insight into the variability of the program’s results and the nuances that characterized their implementation in the territories.

Finally, it is necessary to emphasize that this dependence, although a legitimate criticism made by the leaders, was also recognized as a process of adaptation rather than a permanent state, since it was the result of the gradual strengthening of autonomy in communities that had built mechanisms for problem-solving in the face of the state neglect that characterized interactions with representatives of previous governments. Regarding the criticisms of the programs, it is necessary to clarify that, due to their initial stage and long duration, and the lack of specific limitations on their implementation, the beneficiaries in these communities focused on the positive aspects. This was not due to a lack of agency, but rather to the transition from a state of almost complete lack of support to the presence of external actors with a genuine interest in the community’s well-being.

### 3.3 Integrative analysis: from vulnerability to community agency

The documented process demonstrated a substantial transformation in the participating communities, allowing them to transition from conditions of extreme vulnerability to the progressive exercise of community agency. While victim status was seen as a central obstacle to well-being, the community reality was exacerbated by considerable territorial disparities in institutional care.

This fragmented state of state response, marked by functional encampments in some cases and complete absence in others, resulted in the inability to process grief functionally, the deepening of persistent stigmatization, and the loss of basic security. The four emerging themes presented below synthesize the mechanisms through which the PDPs contributed to reversing these conditions, and each is anchored in the testimonies of the leaders and in the specific activities through which the transformation unfolded.

#### 3.3.1 Mechanisms of transformative change

A crucial outcome in this regard is that participation in the Development and Peace Programs enabled the creation of individual, social, and environmental conditions that significantly contributed to promoting community mental health. The categories associated with these programs revealed that the development of specific resources for coping and strengthening resilience led to considerable improvements in psychological and community well-being.

These specific resources for coping and strengthening resilience were not abstract constructs but concrete tools and spaces promoted by the programs. They included: psychosocial accompaniment workshops, in which participants learned to identify and name their emotions; collective memory exercises, such as memory museums, testimonial writing, and community murals; training in conflict mediation and non-violent communication; and peer support networks, in which leaders accompanied other community members in their own processes. A leader from Huila described one of these tools: “*In the workshops they taught us to breathe, to recognize what we felt, to put words to what had happened. Before, we only knew how to keep quiet. Now we know how to talk about it without it destroying us*” (F49PDPHP). Another leader, from Magdalena Centro, described the collective memory exercises: “*We made a mural with the names of the people we had lost. It was painful, but it was also a way of saying: they existed, we remember them, and we are still here. That helped us a lot to move forward together*” (F58PDPMC). By offering tools for individual and collective processing of traumatic experiences during the conflict, the programs enhanced community development from within, extending from the individual to the collective.

The possibility of having safe spaces to express grief, whether verbally, in writing, or through dramatization, proved to be a transcendental factor in the development of alternatives, since a positive approach to mental health and proactivity in the face of reality’s demands were the foundation for achieving behavioral changes. These spaces included, among others, the memory circles in HUIPAZ, the psychosocial accompaniment tables in PDPMC, and the community dialogue workshops in PRODEPAZ. In this sense, the programs offered spaces and specialized counseling for people to transform their relationships with each other and with objects and phenomena in reality. Through progressively replenished trust, participants’ interactions with themselves, with others, and with institutions improved. A leader from Antioquia summarized this process: “*The program gave us a space to be heard. That was the beginning. When you feel heard, you start to trust. And when you trust, you start to act*” (M47PDP).

#### 3.3.2 Political and organizational dimension

Participation emerged as a fundamental element for political advocacy, as it constituted a conceptual and practical bridge for building common goals aimed at guaranteeing human rights. Here, the programs provided the necessary scaffolding to transform the satisfaction of everyday needs into collective processes with a legal and human foundation that sought to reverse the past lack of transparency. This did not mean that the leaders were delighted, as they reported dissatisfaction related to the overload or instability of efforts, but they also expressed pride and commitment.

This scaffolding materialized in specific training and organizational spaces. In PRODEPAZ, the youth leadership school trained young people in advocacy, project formulation, and public speaking, and promoted their election as community representatives in local governance spaces. In HUIPAZ, the Network of Residents (*Red de Pobladores*) articulated community leaders across municipalities to monitor public policies and to present proposals to local authorities. In PDPMC, the subregional working tables functioned as permanent spaces for dialogue between communities, institutions, and, in some cases, former combatants. A young leader from Antioquia described the youth leadership school: “*The youth school taught us that we didn’t have to wait until we were older to participate. We learned to speak in public, to prepare a proposal, to ask for accountability. Now I am a representative in the municipal youth council, and I am there because of what I learned*” (M25PDP).

This topic synthesized the communities’ feelings regarding political influence and action, which were recognized as mechanisms for unifying community voices and as a vehicle for empowering participatory processes with a democratic perspective. Compared to the distrust and rejection of politics as a reflection of institutionality, the impact of the programs was reflected in the coordination of individuals, organizations, and communities to control vital decisions.

Another key aspect to highlight was the generational transition, facilitated by youth participation and support mechanisms such as the youth leadership school and the election of young people as community representatives. This generational transition was not accidental: it was the result of deliberate program strategies that included scholarships for young leaders, mentoring by older leaders, and the creation of youth platforms in all three regions. This transformation was especially valuable and celebrated by the leaders, as they believe it is the basis for ensuring the systematization of contributions and the continuity of initiatives aimed at strengthening collective well-being. An older leader from Magdalena Centro expressed this: “*It fills me with hope to see the young people taking the lead. We are not going to be here forever. Knowing that they are prepared, that they know how to negotiate, that they are not afraid to speak, that gives me peace*” (F60PDPMC).

#### 3.3.3 Building peace and equity

According to the analysis, the programs fostered strategies and generated guarantees for free expression. This was reflected in the partnership between territorial actors and the fostering of respect for differences and the promotion of equity. In the case of the leaders, they provided testimony of personal transformations and their ability to overcome adverse situations and contribute to decision-making.

The guarantees for free expression were not merely discursive but were embedded in the programs’ operational methodologies. These included the explicit rule that all voices, including those of victims, former combatants, and critics of the programs, had a place in the dialogue spaces; the use of participatory methodologies that ensured that women, youth, and ethnic minorities were heard; and the accompaniment of psychosocial teams trained to prevent re-victimization during these exchanges. A leader from Huila described how this worked in practice: “*In the dialogue spaces, everyone had a turn. It didn’t matter if you were a victim, a neighbor, or even a former combatant. Everyone had to listen. That is how we learned to respect each other, even when we disagreed*” (M52PDPHP).

A critical outcome in this regard was the emergence of dialogue as a central element in conflict resolution processes. Furthermore, this category was associated with values such as tolerance and respect for differences, two fundamental qualities for uniting efforts and finding points of shared worldviews. Here, the programs achieved a fundamental result by transferring technical learning to community dynamics, where dialogue became a guiding principle for relationships. A leader from Antioquia described this transfer: “*Now, in the Community Action Board, when there is a conflict, the first thing we do is call a meeting and talk. We learned that from the program. Before, we would go to the mayor’s office or to the armed group. Now we solve it among ourselves*” (M47PDP). This transformation was especially significant in a context where, as previously documented, dialogue had been systematically replaced by coercion and silence. The programs did not eliminate conflict, which would have been an unrealistic expectation, but they provided communities with the tools and the legitimacy to manage it without resorting to violence or external arbitration.

#### 3.3.4 Projections and sustainability

The need to continue working on truth-building is recognized as a fundamental step toward reconciliation and sustainable peacebuilding. Furthermore, the new forms of community interaction with external factors have shown that peacebuilding cannot depend exclusively on communities and their internal resources. These strengths, although fundamental, require institutional support and consistent, coordinated intervention for their implementation and sustainability.

The leaders identified several specific challenges for sustainability. First, the continuity of the dialogue spaces depended on external funding, which was often unstable. Second, the generational transition, while celebrated, also raised concerns about the loss of institutional memory and the risk that younger leaders might not have the same access to training and accompaniment. Third, the relationship with local authorities remained fragile, as it depended on the political will of each administration. A leader from Huila expressed these concerns: “*The program taught us a lot, but we are worried about what will happen when it ends. The mayor changes every four years, and each one has different priorities. We need the program to keep supporting us, but we also need the institutions to commit for the long term*” (F40PDPHP).

In this sense, the codes and their positive significance showed that the communities recognized the training, support, and organizational strengthening processes as irreplaceable opportunities for growth. A critical view of these supports was also drawn, as those interviewed stated that the progress achieved did not constitute, from their perspective, definitive solutions, but rather the foundation for more profound changes. Regarding these possible future transformations, the analysis showed that communities must continue to build alternatives to strengthen their well-being, forge bonds of shared responsibility with local authorities, and include other local stakeholders in their development plans. A leader from Magdalena Centro summarized this projection: “*What we have achieved is important, but it is not enough. We need the program, the mayor, the governor, the national government, all of us to continue working together. Peace is not built in a day, and it is not built alone*” (M39PDPMC).

#### 3.3.5 Regional variations in the transformation process

Although the analysis identified common patterns of psychosocial transformation across the three programs, the narratives also revealed important regional differences that qualify the general findings. These differences are consistent with the distinct historical trajectories, territorial dynamics, and conflict configurations described in Section 2.1.

In PRODEPAZ (eastern Antioquia), the transformation was most visible in the strengthening of citizen and political participation. Leaders from this region emphasized the role of the youth platform and the leadership school in enabling them to engage with municipal authorities and to influence territorial planning. This emphasis is consistent with the program’s operational strategy, which prioritizes organizational and institutional strengthening and articulation with territorial planning processes.

In HUIPAZ (Huila and Amazonian Foothills), the transformation was most visible in the recovery of voice and the processing of traumatic experiences. Leaders from this region emphasized the emotional and symbolic dimensions of the process, particularly the workshops on memory and the Network of Residents (*Red de Pobladores*). This emphasis is consistent with the program’s focus on peacebuilding, human rights, and the construction of a contextualized model of sustainable human development.

In PDPMC (Magdalena Centro), the transformation was most visible in the rebuilding of trust and the collaborative management of conflicts. Leaders from this region emphasized the role of the subregional working tables in bringing together actors who had previously been separated by fear and mistrust. This emphasis is consistent with the program’s dual nature as both a corporate entity and a psychosocial and community intervention program, and with its strategic lines on culture of peace and reconciliation.

These regional variations suggest that the psychosocial transformation promoted by the PDPs is not a homogeneous process but one that is shaped by the specific socio-territorial conditions in which each program operates. Future research should adopt a comparative longitudinal design to examine how these conditions condition the trajectories and sustainability of the transformations.

## 4 Discussion

This study set out to analyze the psychosocial impact of the Development and Peace Programs from the perspective of community leaders in three regions of Colombia. Its findings contribute to the field of community psychology by documenting how community-based peacebuilding programs can foster psychosocial transformation in contexts of prolonged violence. From this perspective, and particularly from the Latin American tradition inaugurated by Martín-Baró (1994) and continued by Lykes (2001), as well as the contributions of Sanabria Martínez (2022), the recovery of the social fabric in contexts of political violence requires the reconstruction of collective voice, the strengthening of community agency, and the transformation of the subjective and relational conditions that sustain violence and exclusion.

The findings of this study confirm this tradition, insofar as they show that the transformation from vulnerability to agency is a process that requires deliberate pedagogical and psychosocial scaffolding. The programs studied did not limit themselves to providing material resources or technical assistance; they created safe spaces for emotional expression, promoted collective memory exercises, and trained communities in dialogue and conflict mediation. This finding coincides with Sanabria Martínez (2022) argument that the right to free expression, exercised in a context of respect for fundamental rights, is a critical factor in the recovery of the social fabric. It also extends this argument by showing that free expression does not emerge automatically but must be cultivated through specific methodologies and sustained accompaniment.

Furthermore, our findings show that community participation and empowerment processes, as argued by Moreira and Reis Fonseca (2024) and Akhter et al. (2022), do indeed assert the role of individuals and groups in proposing concrete steps to strengthen their well-being. However, this study broadens this conception by considering that participation is not a homogeneous phenomenon. The leaders distinguished between preexisting community participation (constrained by fear and coercion) and participation in the programs, which became active, committed, and effective. This distinction suggests that community psychology should pay closer attention to the different registers of participation and to the conditions under which each becomes possible.

Moreover, the findings obtained here confirm that psychological well-being is a complex category that cannot be reduced to the presence or absence of symptoms, as Pérez Valdivia et al. (2024) have argued. In this study, well-being was described by the leaders not in clinical terms but in relational and collective terms, such as the ability to speak without fear, to sit down with those who were once seen as enemies, to decide together, and to project a shared future. This finding aligns with the broader literature on collective well-being and social cohesion in post-conflict settings (Lira, 2010; Pérez Gamboa et al., 2023; Vonhm, 2025) and suggests that psychosocial interventions should be evaluated not only by their clinical outcomes but also by their capacity to rebuild the relational fabric of communities.

Now, the analysis of the narratives showed that, before the intervention of the Development and Peace Programs, communities faced severe limitations in deploying their resources and capabilities. This finding coincides with multiple studies that have documented these limitations as a direct consequence of the armed conflict, both in rural and urban areas (Ocampo Talero et al., 2022; Rettberg and Dupont, 2025). It also coincides with Duarte Cuervo (2023) observation that these consequences were not only caused by the direct effect of the conflict but also by the difficulty of health professionals in offering a coherent response to demands for psychological help. In our study, this difficulty was expressed in the absence of specialized psychosocial care in the territories prior to the programs, which deepened the effects of trauma and contributed to the chronicity of fear and anxiety.

Along these lines, the study by Gómez-Cano et al. (2024) on the psychosocial impact of the conflict found that women were particularly exposed to this lack of attention, being re-victimized by the absence or inefficiency of support structures. Our findings confirm this observation and extend it by showing that women were not only disproportionately affected but also disproportionately active in the processes of transformation. In all three programs, women constituted the majority of the leaders interviewed and were often the ones who initiated processes of collective memory, mutual support, and dialogue. This finding resonates with the broader literature on women’s roles in peacebuilding (Bendavid et al., 2021) and suggests that psychosocial interventions should recognize and strengthen women’s leadership as a central axis of community recovery.

Specifically, in this study, the psychological effects generated by armed violence manifested themselves in problems in social ties, characterized by permanent feelings of fear and anxiety that impeded participation in collective construction processes. Fear and anxiety, although initially adaptive responses to situations perceived as threatening, when chronically prolonged in contexts of armed conflict, generate dysfunctional identities where emotions are repressed, hindering the construction of relationships that promote individual and collective well-being (Moreno-Chaparro et al., 2022). Our findings confirm this characterization and add a nuance: the leaders described fear not only as an individual emotional state but also as a relational and community condition, one that shaped the ways in which people related to each other, to institutions, and to the territory itself. This suggests that psychosocial interventions should address fear not only at the individual level but also at the level of community relationships and territorial belonging.

The specialized literature corroborates that these traumatic experiences of an emotional and relational nature often face serious difficulties in their functional processing (Jain et al., 2022). Thus, the results allow us to infer that the difficulties in emotional regulation could be due to the systematic use of avoidance as a predominant coping strategy. This inference coincides with the findings of Duarte Cuervo (2023), Gómez-Cano et al. (2024), and Pérez Gamboa et al. (2023), who have documented the prevalence of avoidance and the absence of specialized psychosocial care in populations of victims of the conflict. However, our findings also suggest that avoidance is not an irreversible condition. The programs studied were able to interrupt the cycle of avoidance by offering safe spaces for expression and by training communities in emotional regulation and conflict mediation. This finding has practical implications for the design of psychosocial interventions, as it suggests that the key is not only to provide care but also to create the conditions under which care can be received and transformed into collective action.

The findings of this study also contribute to current debates on peacebuilding and reconciliation. In the field of peace psychology and transitional justice, scholars have emphasized the importance of processing traumatic experiences, rebuilding trust, and fostering collective agency as prerequisites for reconciliation (Lambourne, 2025; Lira, 2010; Pérez Valdivia et al., 2024; Pérez Gamboa et al., 2023). Our findings confirm these arguments and provide empirical evidence on how these processes unfold in practice. Specifically, they show that reconciliation is not a single event but a gradual process that begins with the creation of safe spaces for expression, continues with the rebuilding of trust, and culminates in the exercise of collective agency in public affairs.

According to Pérez Gamboa et al. (2023), based on the study of the representational processes of ex-combatants around forgiveness, peacebuilding, and reconciliation, an intricate web of meanings and symbolisms is produced that goes back to the history of the conflict itself and that frequently reduced the Agreement to a moment that would suddenly erase the causes of the conflagration. Our findings support this critique and extend it by showing that communities themselves do not experience reconciliation as a sudden event but as a long and uneven process, marked by advances and setbacks, and requiring continuous accompaniment. This finding resonates with Lederach (2023) concept of “slow peace” and with Kurtenbach (2025) argument that peace is a normative and territorial process conditioned by local differences.

Furthermore, the study contributes to the debate on the role of communities as active agents of their own transformation, rather than passive recipients of external interventions (Lederach, 1997; Mac Ginty, 2014; Autesserre, 2021). The findings show that the communities studied did not wait for the programs to begin their transformation. They had already developed organic forms of resistance, mutual support, and survival, as documented by Courtheyn (2022) and Lederach (2023). The programs, in this sense, did not create agency ex nihilo but strengthened preexisting community resources. This finding has important implications for the design of peacebuilding interventions: it suggests that external actors should begin by recognizing and strengthening the capacities that already exist in communities, rather than imposing external models or assuming that communities are empty vessels to be filled.

Finally, the findings contribute to the debate on the sustainability of peacebuilding interventions. The leaders interviewed recognized that the programs’ contributions were significant but not definitive, and that their sustainability depended on institutional support and on the communities’ own capacity to maintain the processes over time. This finding coincides with Rettberg and Dupont (2025) argument that PDPs are civil society organizations that facilitate processes but cannot replace the State in guaranteeing rights and services. It also resonates with Cairo et al. (2024) and Ríos (2023) analyses of the challenges of territorial peace in Colombia, which have emphasized the need for coordinated, long-term, and context-sensitive interventions.

### 4.1 Limitations and future directions

Despite the significant findings of this study, it is necessary to acknowledge certain limitations. While this research offers a cross-sectional view of three distinct regions (Huila, Antioquia, and the Middle Magdalena), it does not delve into the historical and sociopolitical particularities that characterized the armed conflict in each territory before the intervention of the programs. The dynamics of violence, the armed actors involved, and the initial state of community mental health likely presented specific racial and identity-based expressions in these regions, which may have influenced the diverse trajectories of psychosocial transformation. Although we have documented some regional variations in Section 3.3.5, a more detailed analysis of these differences was beyond the initial scope of the research. This limitation suggests that future studies should adopt a comparative longitudinal approach to elucidate how specific territorial histories condition the success of peacebuilding programs.

It is also important to note that the data were collected and analyzed with a retrospective approach, capturing leaders’ perceptions of past experiences that often do not conform to rigid chronological periods. Consequently, details about real-time interactions between communities, peace programs, and armed actors were not explicitly collected, as they were not part of the main objectives of the research. During the editorial process and manuscript refinement, we conducted a secondary review of field notes and recordings to address these nuances. However, the available data did not allow for a definitive characterization of these complex territorial tensions. This limitation underscores the need for future research to incorporate real-time ethnographic monitoring, which will allow for a deeper exploration of the changing relationships between community initiatives and the evolving dynamics of local conflict. Implementing a multimodal study that incorporates diverse qualitative designs and the analysis of historical data could provide a more comprehensive understanding of the structural challenges that persist in the context of the Colombian post-conflict.

Furthermore, while the study included leaders with diverse perspectives, including some who expressed criticisms of the programs, the sample was limited to leaders who had maintained a sustained relationship with the PDPs. This may have introduced a selection bias that overrepresents positive experiences. Future research should include the voices of community members who did not participate in the programs, as well as those who withdrew or were excluded, in order to provide a more complete picture of the programs’ reach and limitations.

## 5 Conclusion

The study concludes that, while the programs laid the fundamental foundation for psychosocial recovery in communities affected by violence, it is critical to recognize that these interventions do not function on their own as absolute solutions. Therefore, in the future, programs must ensure that communities are prepared to continue building alternatives that promote well-being. Furthermore, in relation to historical relationships with government entities, it is essential that these alternatives and partnerships be developed in co-responsibility with the State, which must maintain its commitment to guaranteeing comprehensive care and support to those who require it.

On the other hand, it is essential to recognize the central role of communities and victims of conflict in building opportunities for resolving everyday problems, which includes developing means to strengthen well-being and achieve peace. The lessons learned through these processes represent significant contributions to understanding community recovery mechanisms in post-conflict contexts. Therefore, future studies should delve deeper into these processes to inform not only local programs and initiatives, but also the development and implementation of public policies that transcend the identified weaknesses.

In conclusion, the programs studied facilitated transformative processes that allowed communities to transition from states of paralysis due to trauma to scenarios of community agency, where the recovery of voice, meaningful participation, and rebuilding trust emerged as fundamental pillars for collective mental health. These findings provide valuable elements for the design of psychosocial interventions in contexts affected by violence, highlighting the importance of approaches based on strengthening community capacities and rebuilding local power.

## Data Availability

The datasets presented in this study can be found in online repositories. The names of the repository/repositories and accession number(s) can be found in the article/supplementary material.
